# Analysis of the short-term prognosis and risk factors of elderly acute kidney injury patients in different KDIGO diagnostic windows

**DOI:** 10.1007/s40520-019-01261-z

**Published:** 2019-08-13

**Authors:** Qinglin Li, Zhi Mao, Pan Hu, Hongjun Kang, Feihu Zhou

**Affiliations:** Department of Critical Care Medicine, The First Medical Center, Chinese PLA General Hospital, 28 Fuxing Road, Beijing, 100853 China

**Keywords:** Acute kidney injury, AKI diagnosis window, Very elderly, Short-term mortality

## Abstract

**Background and aims:**

Follow-up observation was performed on elderly acute kidney injury (AKI) patients to analyze the short-term prognosis and risk factors of AKI patients in the 48-h diagnostic window and 7-day diagnostic window of the Kidney Disease: Improving Global Outcomes (KDIGO) guidelines.

**Methods:**

Inpatients aged ≥ 75 years in the geriatric ward of the People’s Liberation Army General Hospital, China, between January 2007 and December 2015 were selected as the research subjects. According to two diagnostic criteria in the KDIGO guidelines, patients were divided into a 48-h diagnostic window group and a 7-day diagnostic window group. The medical data of the patients were divided into the death group and the survival group for analysis based on the survival condition of the patients after 90 days of AKI. Factors that affected the 90-day survival of patients in the 48-h diagnostic window and 7-day diagnostic window groups were analyzed using multivariate Cox regression.

**Results:**

During the follow-up period, a total of 652 patients were enrolled in this study. Among them, 623 cases were men, accounting for 95.6% of the patients. The median age was 87 (84–91) years. According to the KDIGO staging criteria, there were 308 (47.2%) cases in AKI stage 1, 164 (25.2%) cases in stage 2, and 180 (27.6%) cases in stage 3. Among the 652 patients, 334 (51.2%) were diagnosed with AKI based on the 48-h diagnostic criteria window, and 318 (48.8%) were diagnosed with AKI based on the baseline 7-day diagnostic criteria. The 90-day mortality of AKI patients was 42.5% in the 48-h diagnostic window and 24.2% in the 7-day diagnostic window. The multivariate Cox analysis results showed that low mean arterial pressure (HR = 0.966; *P *< 0.001), low serum prealbumin level (HR = 0.932; *P *< 0.001), infection (HR = 1.448; *P *= 0.047), mechanical ventilation (HR = 1.485; *P *= 0.038), high blood urea nitrogen (BUN) level (HR = 1.026; *P *< 0.001), blood magnesium level (HR = 2.560; *P *= 0.024), and more severe AKI stage (stage 2: HR = 3.482; *P *< 0.001 and stage 3: HR = 6.267; *P *< 0.001) were independent risk factors affecting the 90-day mortality of elderly patients in the 48-h diagnostic window, whereas low body mass index (HR = 0.851; *P *< 0.001), low mean arterial pressure (HR = 0.980; *P *= 0.036), low serum prealbumin level (HR = 0.950; *P *= 0.048), low serum albumin level (HR = 0.936; *P *= 0.015), high BUN level (HR = 1.046; *P *< 0.001), and more severe AKI stage (stage 2: HR = 4.249; *P *= 0.001 and stage 3: HR = 9.230; *P *< 0.001) were independent risk factors affecting the 90-day mortality of elderly patients in the 7-day diagnostic window.

**Conclusions:**

The clinical differences of AKI and risk factors for 90-day mortality in elderly AKI individuals vary depending on the definition used. An increment of Scr ≥ 26.5 μmol/L in 48 h (48-h KDIGO window) alone predicts adverse clinical outcomes.

## Introduction

Acute kidney injury (AKI) is a common critical and severe disease that occurs in various clinical departments. There is rapid decline of kidney function within a short time period (several hours to several days) and the retention of metabolic waste resulting from multiple causes. The diagnosis of AKI depends on an increase in serum creatinine (Scr) and a decrease in the urine volume. The proposed AKI concept replaces acute renal failure (ARF), which has been used for many years. The incidence of AKI is high and shows an increasing year-on-year trend. The annual worldwide incidence of AKI is approximately 2100 people/million people [1], and the number of deaths every year reaches 2 million [2]; of which, in-hospital deaths account for 20–25% [3]. In addition, the mortality of severe AKI patients during dialysis treatment is greater than 50% [3]. A recent meta-analysis report that included more than 700 studies showed that the prognosis of AKI does not show significant improvement [4], suggesting that AKI is an important disease that severely dangers the health, life, and safety of people [5, 6].

To perform an early diagnosis of AKI and improve the prognosis, the concept and diagnostic criteria of AKI underwent three main changes: the Risk, Injury, Failure, Loss of kidney function, and End-stage kidney disease (RIFLE) criteria in 2002 [7], the Acute Kidney Injury Network (AKIN) criteria in 2004 [8], and the Kidney Disease: Improving Global Outcomes (KDIGO) criteria in 2012 [9]. The AKI guidelines in KDIGO increased the time concept and defined AKI as an increase in Scr ≥ 0.3 mg/dl (26.5 μmol/L) within 48 h or an increase in Scr over the baseline value by 1.5-fold within 7 days.

Currently, there are few studies regarding the short-term prognosis and clinical manifestations of elderly patients in the 48-h and 7-day time windows of the KDIGO guidelines [10]. Therefore, we retrospectively analyzed clinical information of elderly inpatients in the People’s Liberation Army General Hospital, China. The retrospective cohort study was performed using 90 days after the development of AKI as the observation point. Patients in the 48-h time window and 7-day time window were analyzed to understand the mortality, clinical characteristics, and factors affecting the prognosis of patients in different AKI diagnostic windows.

## Patients and methods

### Study design, setting, and population

We retrospectively analyzed clinical data from very elderly patients (≥ 75 years of age) who were admitted to the Geriatrics Department of the Chinese PLA General Hospital in Beijing, China, between January 1, 2007, and December 31, 2015. The study design was approved by the Clinical Ethics Committee of the Chinese PLA General Hospital, and each patient provided informed written consent. All admissions were screened and evaluated for AKI, categorizing them according to the Kidney Disease Improving Global Outcomes (KDIGO) criteria. AKI patients were divided into the 48-h diagnostic window group and the 7-day diagnostic window group based on the KDIGO diagnostic criteria. The medical data of patients in the different diagnostic windows were divided into the survival group and the death group for analysis.

### Definitions

For the AKI definition and diagnostic criteria, the Scr diagnostic criteria defined in the KDIGO AKI guidelines were adopted [9]: (1) increase in Scr higher than 26.5 μmol/L within 48 h or (2) increase in Scr by more than 1.5-fold above the baseline value (confirmed or suspected) within 7 days. The AKI classification criteria used the Scr classification criteria defined by the KDIGO guidelines, and there were three stages. The CKD definition and classification criteria used the CKD criteria in the KDIGO guidelines [11]. The baseline value of Scr was the Scr measurement value of patients obtained in a stable state within 3 months of AKI onset [12]. The Scr peak value was the maximum value of Scr detected during the disease course of AKI. The estimated glomerular filtration rate (eGFR) was determined with the 2009 Chronic Kidney Disease Epidemiology Collaboration (CKD-EPI) equation [13]. Oliguria was defined as urinary output, 400 mL/24 h.

### Data collection

The general conditions [name, age, gender, and body mass index (BMI)], history of underlying diseases [mainly coronary heart disease, hypertension, diabetes mellitus (DM), chronic obstructive pulmonary disease (COPD), and chronic kidney disease (CKD)], clinical medication conditions, AKI etiology, accompanying conditions upon AKI onset (AKI diagnosis time, mechanical ventilation, urine volume, dialysis, and mean arterial pressure), and laboratory examination indicators including Scr, Scr peak value, blood urea nitrogen (BUN), serum uric acid, serum prealbumin, serum albumin, blood magnesium, blood calcium, blood phosphorus, and hemoglobin results of the patients were recorded.

### Exclusion criteria

We excluded patients younger than 75 years, those with previously diagnosed CKD, those who stayed in the hospital for < 48 h, those who underwent fewer than two Scr examinations, those with missing or incomplete medical histories, and those who died early within 48 h after admission.

### Statistical analysis

Statistical analyses were performed using SPSS 17.0 statistical software. Continuous variables that conformed to a normal distribution were expressed as $\bar{x}$ ± s, and comparisons between two groups were performed using a *t* test. Continuous variables that did not conform to a normal distribution were expressed as the median value (M) and the inter-quartile range (25–75%), and comparisons between two groups were performed using the Mann–Whitney *U* test. Count data were expressed as *n* (%) and univariate analysis was performed using the Pearson *X*^2^ test or Fisher’s exact test. For multivariate analyses, variables that had significant differences in the univariate analyses were included in the multivariate analyses using the forward method, and multivariate analyses were performed using the Cox survival analysis. *P *< 0.05 was considered to be statistically significant.

## Results

### The general condition of AKI in elderly patients

**Fig. 1:**
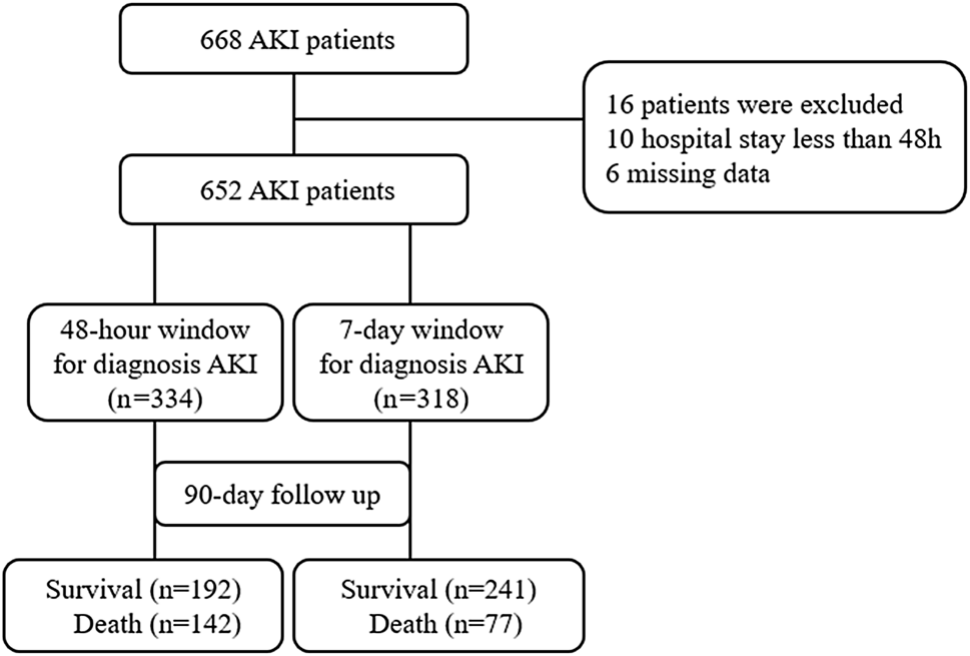
Flow chart of patient inclusion and exclusion. *AKI* acute kidney injury, *CKD* chronic kidney disease, *SCr* serum creatinine

Between January 2007, and December 2015, a total of 3464 very elderly patients were admitted to the Geriatrics Department. During the study period, a total of 668 elderly people developed AKI. After excluding 10 AKI patients who had a hospitalization time < 48 h and 6 AKI patients who had incomplete data, a total of 652 elderly AKI patients who met the criteria were eventually enrolled for analysis. The median age was 87 (84–91) years. There were 623 men, accounting for 95.6% of patients. According to the KDIGO diagnostic criteria, a total of 334 (51.2%) patients were diagnosed with AKI based on the 48-h diagnostic criteria, and 318 (48.8%) patients were diagnosed with AKI based on the baseline values within 7 days. The 90-day mortality in the follow-up after development of AKI was 33.6% (219/652). The mortality rates in the 48-h diagnostic window and 7-day diagnostic window were 42.5% (142/334) and 24.2% (77/318), respectively. The study flow chart is presented, as shown in Fig. 1.

### General conditions and clinical characteristics of patients after the development of AKI in the 48-h diagnostic window group

As shown in Table 1, the general conditions of patients in the survival group and the death group did not have statistically significant differences regarding age (median age: 87 vs 87 years, *P *= 0.515), gender (95.3% vs 93.0%, *P *= 0.358), or the history of accompanying underlying diseases (coronary disease *P *= 0.713, hypertension *P *= 0.410, COPD *P *= 0.821, and DM *P *= 0.454). The BMI of patients in the survival group was higher than that in the death group (23.5 ± 3.4 vs 22.4 ± 2.9 kg/m^2^, *P *= 0.002), the baseline Scr of patients in the survival group was higher than that in the death group (76.0 vs 64.0 μmol/L, *P *< 0.001), and the eGFR of patients in the survival group was lower than that in the death group (77.3 vs 83.8 mL/min/1.73 m^2^, *P *< 0.001). The differences were all statistically significant (Table 1).

**Table 1:** Comparisons of the clinical characteristics between elderly survivors and non-survivors with 48-h window AKI

Characteristic	48-h window AKI patients (*n* = 334)	Non-survivors (*n* = 142)	Survivors (*n* = 192)	*P*
Age (years)	87 (84–90)	87 (83–90)	87 (84–90)	0.515
Male	315 (94.3)	132 (93.0)	183 (95.3)	0.358
BMI (kg/m^2^)	23.0 ± 3.2	22.4 ± 2.9	23.5 ± 3.4	0.002
Comorbidity				
Coronary disease	255 (76.3)	107 (75.4)	148 (77.1)	0.713
Hypertension	243 (72.8)	100 (70.4)	143 (74.5)	0.410
COPD	233 (69.8)	100 (70.4)	133 (69.3)	0.821
Diabetes	131 (39.2)	59 (41.5)	72 (37.5)	0.454
Baseline Scr (μmol/L)	70.0 (60.0–82.0)	64.0 (52.0–75.0)	76.0 (63.0–84.8)	< 0.001
Baseline eGFR (ml/min/1.73^2^)	80.1 (71.8–86.0)	83.8 (77.9–91.0)	77.3 (69.2–83.8)	< 0.001
Etiology of AKI				
Infections	141 (42.2)	76 (53.5)	65 (33.9)	< 0.001
Hypovolemia	80 (24.0)	36 (25.4)	44 (22.9)	0.606
Cardiovascular events	55 (16.5)	16 (11.3)	39 (20.3)	0.028
Nephrotoxicity	22 (6.6)	7 (4.9)	15 (7.8)	0.294
Surgery	31 (9.3)	7 (4.9)	24 (12.5)	0.018
Others	5 (1.5)	0	5 (2.6)	0.018
Parameter at the time of AKI diagnosis				
MAP (mmHg)	77 ± 15	71 ± 13	81 ± 14	< 0.001
Oliguria	24 (7.2)	18 (12.7)	6 (3.1)	0.001
Dialysis	7 (2.1)	5 (3.5)	2 (1.0)	0.118
MV	152 (45.5)	98 (69.0)	54 (28.1)	< 0.001
Laboratory results at the time of AKI diagnosis				
Scr (μmol/L)	131.3 (116.0–153.0)	141.9 (124.8–164.3)	124.4 (112.6–141.0)	< 0.001
Peak Scr (μmol/L)	152.1 (123.7–227.6)	207.9 (157.0–308.7)	134.1 (115.7–160.0)	< 0.001
BUN (mmol/L)	13.9 (9.3–22.7)	21.2 (14.5–30.7)	10.8 (7.6–15.8)	< 0.001
Uric acid (μmol/L)	361.4 (292.9–459.0)	404.5 (306.5–514.6)	340.8 (286.2–424.8)	0.001
Prealbumin (g/L)	168.0 (131.0–212.0)	140 (109–181)	189 (150–230)	< 0.001
Albumin (g/L)	33.5 ± 5.3	31.1 ± 4.8	35.2 ± 5.0	< 0.001
Magnesium (mmol/L)	0.9 (0.8–1.0)	0.9 (0.8–1.0)	0.9 (0.8–0.9)	0.018
Calcium (mmol/L)	2.2 (2.0–2.3)	2.2 (2.0–2.3)	2.2 (2.0–2.3)	0.680
Phosphate (mmol/L)	1.2 (0.9–1.5)	1.3 (1.0–1.6)	1.1 (0.9–1.4)	0.003
Hemoglobin (g/L)	113 ± 23	106 ± 23	118 ± 20	< 0.001
AKI Stage				< 0.001
1	129 (38.6)	12 (8.5)	117 (60.9)	
2	86 (25.7)	40 (28.2)	46 (24.0)	
3	119 (35.6)	90 (63.4)	29 (15.1)	

Values are *n* (%), mean ± SD or median (inter-quartile range)

*AKI* acute kidney injury, *BMI* body mass index, *COPD* chronic obstructive pulmonary disease, *MAP* mean arterial pressure, *1* *mmHg* 0.133 kPa, *MV* mechanical ventilation, *Scr* serum creatinine, *BUN* blood urea nitrogen

In the analyses comparing the clinical characteristics between these two groups, the death group had a higher percentage of patients with infection (53.5% vs 33.9%, *P *< 0.001), but a lower percentage of patients with cardiovascular events (11.3% vs 20.3%, *P *= 0.028) and surgery (4.9% vs 12.5%, *P *= 0.018). The Scr levels (141.9 vs 124.4 µmol/L, *P *< 0.001), Scr peak values (207.9 vs 134.1 µmol/L, *P *< 0.001), BUN (21.2 vs 10.8 mmol/L, *P *< 0.001), and uric acid levels (404.5 vs 340.8 µmol/L, *P *= 0.001) of patients in the death group upon AKI diagnosis were significantly higher than those in the survival group. The accompanying conditions of blood pressure (71 ± 13 vs 81 ± 14 mmHg, *P *< 0.001), mechanical ventilation (69.0% vs 28.1%, *P *< 0.001), oliguria (12.7% vs 3.1%, *P *= 0.001), hypoalbuminemia (31.1 ± 4.8 vs 35.2 ± 5.0 g/L, *P *< 0.001), low prealbumin level (140 vs 189 g/L, *P *< 0.001), anemia (106 ± 23 vs 118 ± 20 g/L, *P *< 0.001), hypermagnesemia (0.9 vs 0.9 mmol/L, *P *= 0.018), and hyperphosphatemia (1.3 vs 1.1 mmol/L, *P *= 0.003) of patients in the death group significantly increased compared to those in the survival group. Table 1 also shows the relationship between the AKI stage and short-term outcome; the percentage of more severe AKI stages (stage 3: 63.4% vs 15.1% and stage 2: 28.2% vs 24.0%) was higher than that in the survival group. Unsurprisingly, outcomes worsened with more advanced AKI stage (*P *< 0.001 for the three stages).

The multivariate Cox analysis results showed that low mean arterial pressure (HR = 0.966; 95% CI 0.953–0.979; *P *< 0.001), low serum prealbumin level (HR = 0.932; 95% CI 0.900–0.964; *P *< 0.001), infection (HR = 1.448; 95% CI 1.004–2.089; *P *= 0.047), mechanical ventilation (HR = 1.485; 95% CI 1.023–2.155; *P *= 0.038), high BUN level (HR = 1.026; 95% CI 1.013–1.040; *P *< 0.001), blood magnesium level (HR = 2.560; 95% CI 1.132–5.789; *P *= 0.024), and more severe AKI stage (stage 2: HR = 3.482; 95% CI 1.801–6.732; *P *< 0.001 and stage 3: HR = 6.267; 95% CI 3.307–11.874; *P *< 0.001) were independent risk factors affecting the 90-day mortality of elderly patients in the 48-h diagnostic window (Table 2).

**Table 2:** Multivariate Cox proportional hazard model analysis of risk factors for 48-h window AKI mortality

Risk factor	HR	95% CI	*P*
MAP	0.966	0.953–0.979	< 0.001
Prealbumin level	0.932	0.900–0.964	< 0.001
Infection	1.448	1.004–2.089	0.047
MV	1.485	1.023–2.155	0.038
BUN level	1.026	1.013–1.040	< 0.001
Magnesium level	2.560	1.132–5.789	0.024
AKI Stage			< 0.001
Stage 1	–	–	
Stage 2	3.482	1.801–6.732	< 0.001
Stage 3	6.267	3.307–11.874	< 0.001

*HR* hazard ratio, *CI* confidence interval, *AKI* acute kidney injury, *MAP* mean aortic pressure, *1* *mmHg* 0.133 kPa, *MV* mechanical ventilation, *BUN* blood urea nitrogen

### General conditions and clinical characteristics of patients after the development of AKI in the 7-day diagnostic window group

As shown in Table 3, the general conditions of patients in the survival group and death group did not have statistically significant differences regarding age (median age: 87 vs 88 years, *P *= 0.676) and gender (96.7% vs 97.4%, *P *= 0.747). The BMI (23.5 ± 3.1 vs 21.9 ± 2.8 kg/m^2^, *P *< 0.001), percentage of an accompanying history of hypertension (79.7% vs 64.9%, *P *= 0.008), and baseline Scr (80.0 vs 66.0 μmol/L, *P *< 0.001) of the patients in the survival group were higher than those in the death group, whereas the eGFR (76.4 vs 82.4 mL/min/1.73 m^2^, *P *< 0.001) was lower than that in the death group, and the differences were statistically significant.

**Table 3:** Comparisons of the clinical characteristics between elderly survivors and non-survivors with 7-day window AKI

Characteristic	7-day window AKI patients (*n* = 318)	Non-survivors (*n* = 77)	Survivors (*n* = 241)	*P*
Age (years)	87 (84–91)	88 (85–90)	87 (84–91)	0.676
Male	308 (96.9)	75 (97.4)	233 (96.7)	0.747
BMI (kg/m^2^)	23.1 ± 3.1	21.9 ± 2.8	23.5 ± 3.1	< 0.001
Comorbidity				
Coronary disease	250 (78.6)	64 (83.1)	186 (77.2)	0.269
Hypertension	242 (76.1)	50 (64.9)	192 (79.7)	0.008
COPD	221 (69.5)	47 (61.0)	174 (72.2)	0.064
Diabetes	103 (32.4)	24 (31.2)	79 (32.8)	0.793
Baseline SCr (μmol/L)	76.0 (65.0–85.0)	66.0 (55.5–77.5)	80.0 (70.0–86.0)	< 0.001
Baseline eGFR (ml/min/1.73^2^)	77.2 (70.6–83.0)	82.4 (75.1–87.9)	76.4 (69.5–80.7)	< 0.001
Etiology of AKI				
Infections	118 (37.1)	40 (51.9)	78 (32.4)	0.002
Hypovolemia	75 (23.6)	17 (22.1)	58 (24.1)	0.720
Cardiovascular events	48 (15.1)	11 (14.3)	37 (15.4)	0.820
Nephrotoxicity	56 (17.6)	5 (6.5)	51 (21.2)	0.003
Surgery	14 (4.4)	2 (2.6)	12 (5.0)	0.349
Others	7 (2.2)	2 (2.6)	5 (2.1)	0.789
Parameter at the time of AKI diagnosis				
MAP (mmHg)	80 ± 13	72 ± 12	82 ± 12	< 0.001
Oliguria	11 (3.5)	7 (9.1)	4 (1.7)	0.005
Dialysis	2 (0.6)	1 (1.3)	1 (0.4)	0.430
MV	88 (27.7)	45 (58.4)	43 (17.8)	< 0.001
Laboratory results at the time of AKI diagnosis				
Scr (μmol/L)	131.6 (119.1–143.0)	136.0 (117.1–150.3)	130.4 (120.0–141.0)	0.193
Peak Scr (μmol/L)	139.4 (124.0–175.3)	238.0 (154.2–341.8)	135.2 (122.2–151.5)	< 0.001
BUN (mmol/L)	11.5 (8.6–19.3)	22.5 (14.6–33.3)	10.1 (7.9–14.9)	< 0.001
Uric acid (μmol/L)	371.1 (284.5–471.5)	418.0 (326.0–553.8)	362.5 (279.4–450.1)	0.001
Prealbumin (g/L)	197 (150–259)	151 (115–191)	211 (164–272)	< 0.001
Albumin (g/L)	35.3 ± 5.5	30.9 ± 4.9	36.7 ± 5.0	< 0.001
Magnesium (mmol/L)	0.9 (0.8–1.0)	1.0 (0.8–1.1)	0.9 (0.9–1.0)	0.393
Calcium (mmol/L)	2.2 (2.1–2.4)	2.2 (2.1–2.4)	2.2 (2.1–2.4)	0.236
Phosphate (mmol/L)	1.2 (1.0–1.4)	1.2 (0.9–1.5)	1.2 (1.0–1.4)	0.775
Hemoglobin (g/L)	112 ± 23	94 ± 20	118 ± 21	< 0.001
AKI Stage				< 0.001
1	179 (56.3)	7 (9.1)	172 (71.4)	
2	78 (24.5)	26 (33.8)	52 (21.6)	
3	61 (19.2)	44 (57.1)	17 (7.1)	

Values are *n* (%), mean ± SD or median (inter-quartile range)

*AKI* acute kidney injury, *BMI* body mass index, *COPD* chronic obstructive pulmonary disease, *MAP* mean arterial pressure, *1* *mmHg* 0.133 kPa, *MV* mechanical ventilation, *Scr* serum creatinine, *BUN* blood urea nitrogen

Regarding the clinical characteristics between these two groups, in the death group, the percentage of infection was high (51.9% vs 32.4%, *P *= 0.002), and the percentage of use of nephrotoxic drugs was low (6.5% vs 21.2%, *P *= 0.003). The Scr peak value (238.0 vs 135.2 µmol/L; *P *< 0.001), BUN (22.5 vs 10.1 mmol/L; *P *< 0.001), and uric acid levels (418.0 vs 362.5 µmol/L, *P *= 0.001) of patients in the death group upon AKI diagnosis were significantly higher than those in the survival group. The presence of accompanying hypotension (72 ± 12 vs 82 ± 12 mmHg, *P *< 0.001), mechanical ventilation (58.4% vs 17.8%, *P *< 0.001), oliguria (9.1% vs 1.7%, *P *= 0.005), hypoalbuminemia (30.9 ± 4.9 vs 36.7 ± 5.0 g/L, *P *< 0.001), low prealbumin level (151 vs 211 g/L, *P *< 0.001), and anemia (94 ± 20 vs 118 ± 21 g/L, *P *< 0.001) of patients in the death group was significantly increased compared to those in the survival group. Table 3 also shows the relationship between the AKI stage and short-term outcome; AKI severity was associated with a significantly higher 90-day mortality (9.1% for stage 1 patients, 33.8% for stage 2, and 57.1% for stage 3). Unsurprisingly, outcomes worsened with more advanced AKI stage (*P *< 0.001 for the three stages).

Multivariate analysis by the Cox model revealed that low BMI (HR = 0.851; 95% CI 0.778–0.931; *P *< 0.001), low mean arterial pressure (HR = 0.980; 95% CI 0.962–0.999; *P *= 0.036), low serum prealbumin level (HR = 0.950; 95% CI 0.902–1.000; *P *= 0.048), low serum albumin level (HR = 0.936; 95% CI 0.887–0.987; *P *= 0.015), high BUN level (HR = 1.046; 95% CI 1.022–1.070; *P *< 0.001), and more severe AKI stage (stage 2: HR = 4.249; 95% CI 1.768–10.215; *P *= 0.001 and stage 3: HR = 9.230; 95% CI 3.943–21.605; *P *< 0.001) were independent risk factors affecting the 90-day mortality of elderly patients in the 7-day diagnostic window (Table 4).

**Table 4:** Multivariate Cox proportional hazard model analysis of risk factors for 7-day window AKI mortality

Risk factor	HR	95% CI	*P*
BMI	0.851	0.778–0.931	< 0.001
MAP	0.980	0.962–0.999	0.036
Prealbumin level	0.950	0.902–1.000	0.048
Albumin level	0.936	0.887–0.987	0.015
BUN level	1.046	1.022–1.070	< 0.001
AKI Stage			< 0.001
Stage 1	–	–	
Stage 2	4.249	1.768–10.215	0.001
Stage 3	9.230	3.943–21.605	< 0.001

*HR* hazard ratio, *CI* confidence interval, *AKI* acute kidney injury, *BMI* body mass index, *MAP* mean aortic pressure, *1* *mmHg* 0.133 kPa, *BUN* blood urea nitrogen

### Comparison with 48-h or 7-day diagnostic window AKI on patient short-term outcomes

**Fig. 2:**
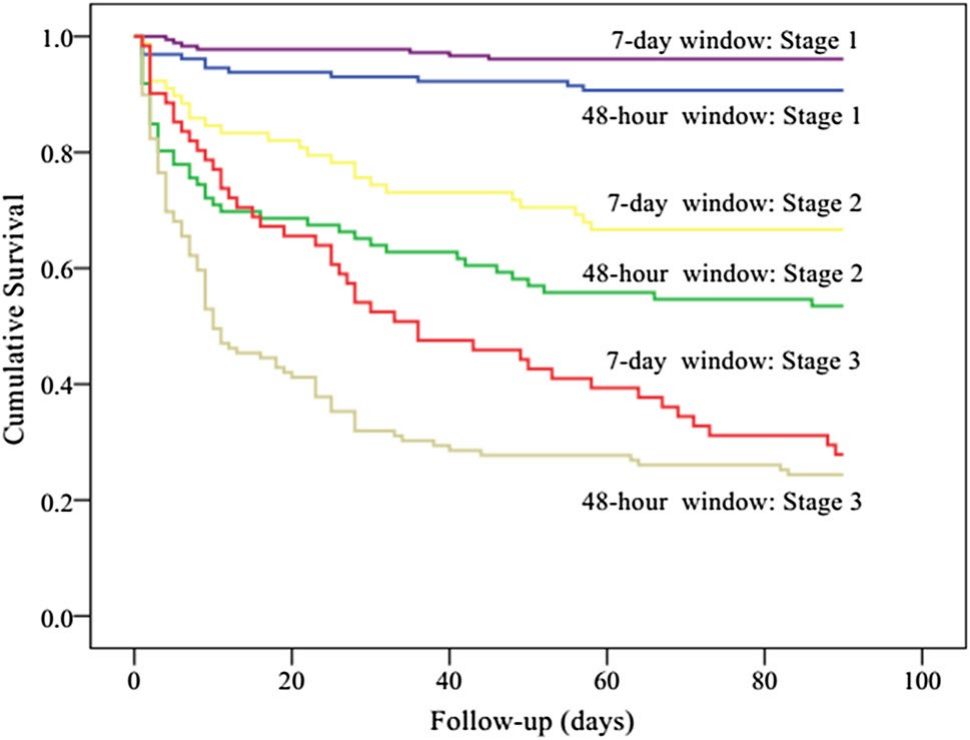
Kaplan–Meier survival curves according to 48-h window and 7-day window AKI at different KDIGO stages (log-rank test: *P *< 0.001). *AKI* acute kidney injury, *KDIGO* kidney disease improving global outcomes

**Fig. 3:**
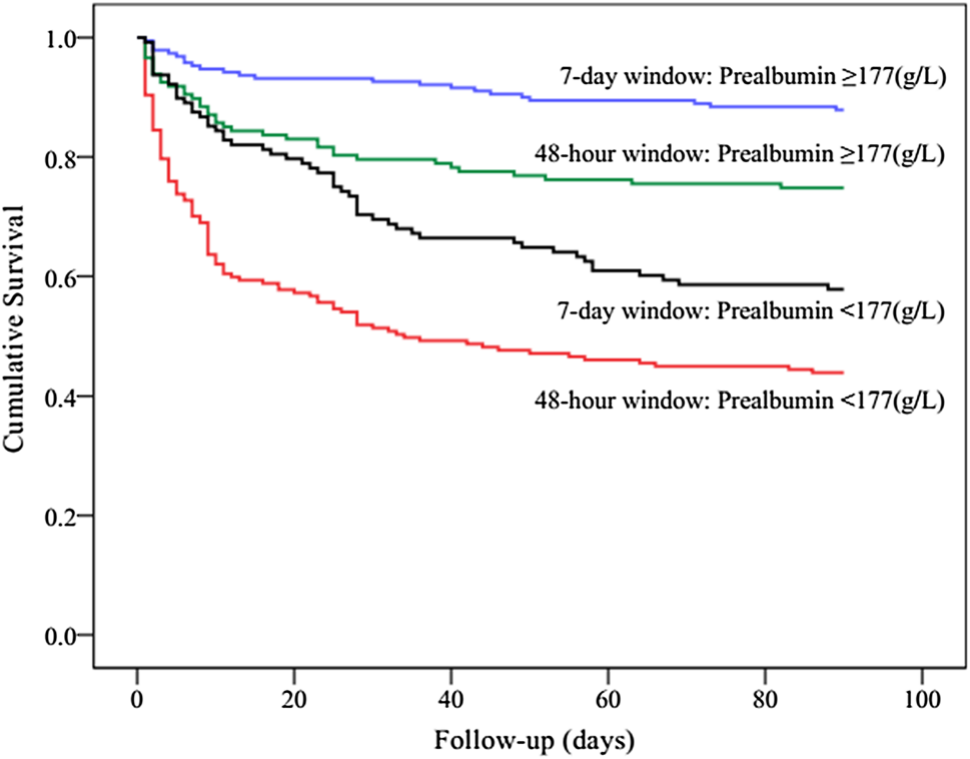
Kaplan–Meier survival curves for 90-day mortality according to prealbumin level, using the receiver operating characteristic analysis as the cutoff point values (177 g/L) (log-rank test: *P *< 0.001)

**Fig. 4:**
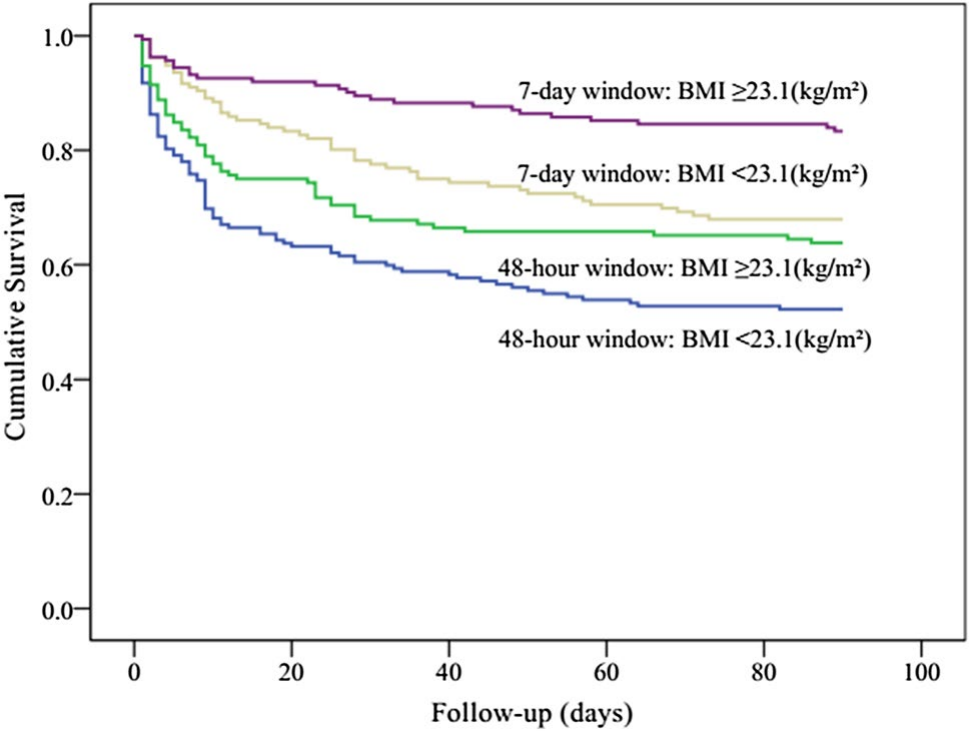
Kaplan–Meier survival curves for 90-day mortality according to BMI, using the receiver operating characteristic analysis as the cutoff point values (23.1 kg/m^2^) (log-rank test: *P *< 0.001). *BMI* body mass index

**Fig. 5:**
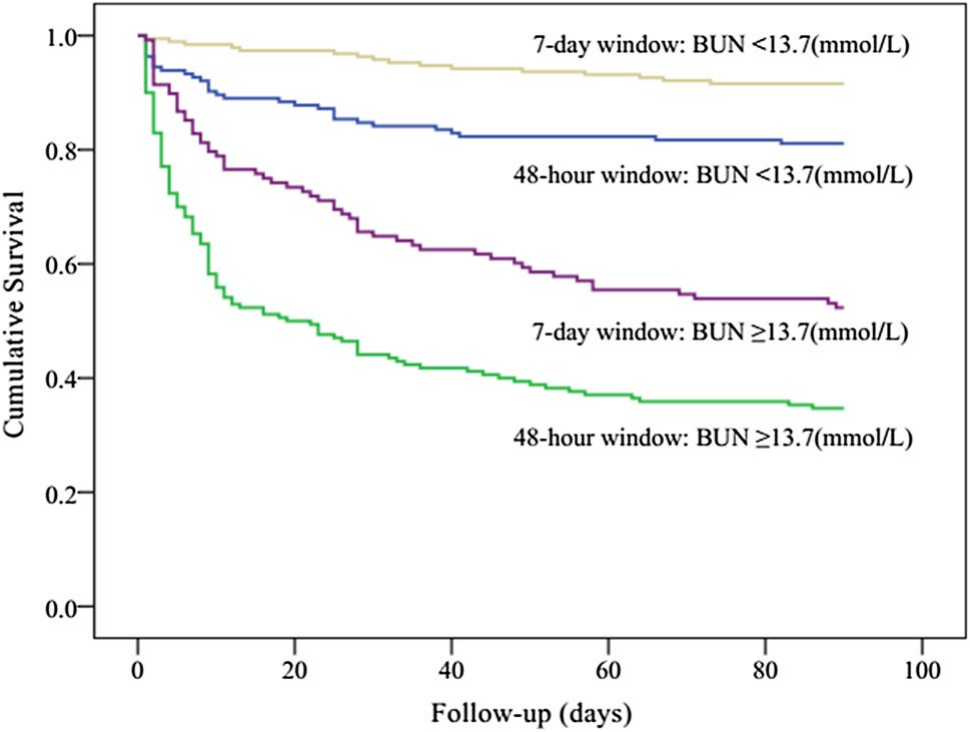
Kaplan–Meier survival curves for 90-day mortality according to BUN, using the receiver operating characteristic analysis as the cutoff point values (13.7 mmol/L) (log-rank test: *P *< 0.001). *BUN* blood urea nitrogen

**Fig. 6:**
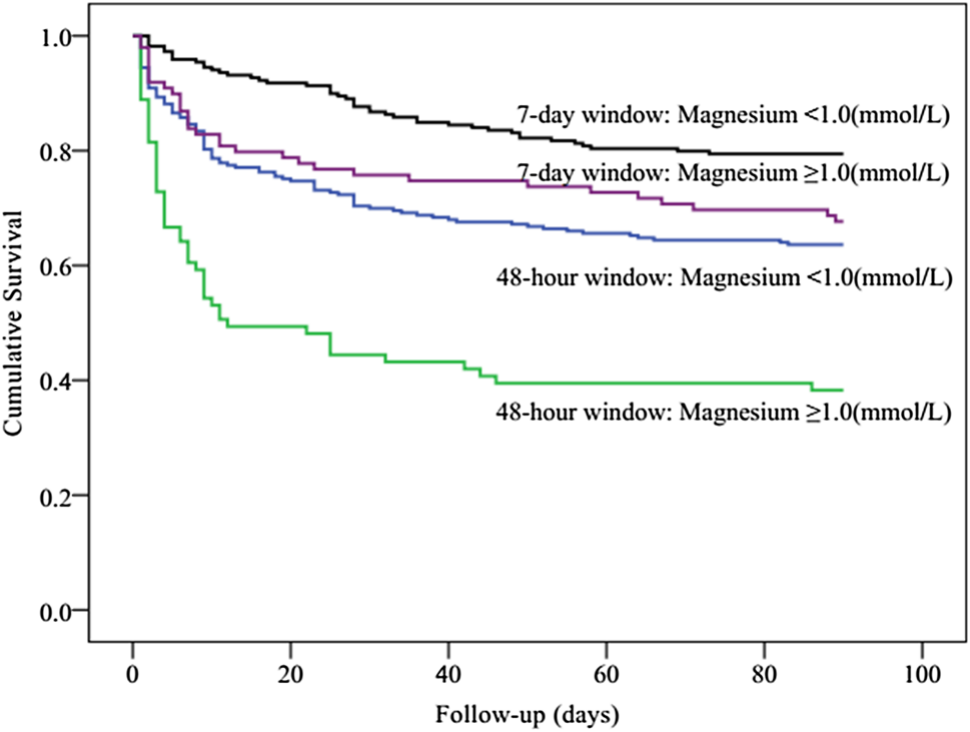
Kaplan–Meier survival curves for 90-day mortality according to magnesium, using the receiver operating characteristic analysis as the cutoff point values (1.0 mmol/L) (log-rank test: *P *< 0.001)

Kaplan–Meier curves showed significant differences in the 90-day mortality between the 7-day diagnostic window and the 48-h diagnostic window groups (log rank *P *< 0.001). The separation of the curves continued throughout the follow-up period, with an increased probability of death during the follow-up with increasing degrees of AKI (log rank *P *< 0.001, Fig. 2). Indeed, in each of the AKI subgroups, the short-term mortality of patients in the 7-day diagnostic window was better than that of patients in the 48-h diagnostic window. According to the Kaplan–Meier plot, patients with prealbumin level ≥ 177 (g/L) had better survival than patients with lower level (log rank *P *< 0.001, Fig. 3). Within the different diagnostic window groups, the 90-day mortality was better in the 7-day diagnostic window than in the 48-h diagnostic window (log rank *P *< 0.001; Fig. 3). Similarly, patients with BMI ≥ 23.1(kg/m^2^) had better survival than patients with lower level (log rank *P *< 0.001, Fig. 4). Patients with BUN level < 13.7 (mmol/L) had better survival than patients with higher level (log rank *P *< 0.001, Fig. 5). Patients with magnesium level < 1.0 (mmol/L) had better survival than patients with higher level (log rank *P *< 0.001, Fig. 6).

## Discussion

This study adopted the KDIGO diagnostic classification system for evaluation of the mortality and related risk factors of elderly patients in 48-h and 7-day diagnostic window groups. The results suggested that the mortality of patients in the 48-h diagnostic window group and the 7-day diagnostic window group were 42.5% and 24.2%, respectively. Low mean arterial pressure, low serum prealbumin level, infection, a need for mechanical ventilation, high BUN level, blood magnesium level, and more severe AKI stage were independent risk factors affecting the short-term mortality of AKI patients in the 48-h diagnostic window, whereas low BMI, low mean arterial pressure, low prealbumin level, low albumin level, high BUN level, and more severe AKI stage were independent risk factors affecting the short-term mortality of AKI patients in the 7-day diagnostic window.

Recently, Sparrowet HG al published a study with 81,651 patients with AKI using KDIGO definition [14]. In this study, patients had a mean age of 65 years (18–107). They defined AKI stage 1a as an absolute increase in Scr of 0.3 mg/dl within 48 h and stage 1b as a 50% relative increase in Scr within 7 days. The incidence of in-hospital AKI was 7.5% for stage 1a, 4.9% for stage 1b, 1.5% for stage 2, and 0.9% for stage 3, incidence of in-hospital mortality was 2.2% for stage 1a, 5.5% for stage 1b, 17.8% for stage 2, and 24.2% for stage 3. The findings of the study demonstrated that patients with AKI stages 1a and 1b experienced clinically meaningful and statistically significant differences for outcomes of hospital length of stay and in-hospital mortality.

There has been a lack of uniform definitions and diagnostic criteria, since the concept of acute renal failure (ARF) was proposed, and as many as 35 definitions have been roughly counted in various literature sources. Because the definition of ARF has not yet reached consensus, its epidemiological study results such as incidence and mortality vary widely, and different study results are difficult to compare. For the early diagnosis of AKI and a reduction of its missed diagnosis rate, the KDIGO guidelines provide the 48-h diagnostic window and the 7-day diagnostic window. However, the following deficiencies remain in the clinical implementation process. First, “abrupt decrease in kidney function” is limited to within 48 h; therefore, theoretically, only those patients who have their Scr value measured more than 2 times within 48 h can be detected. This can be difficult in clinical practice. Next, the starting time of renal replacement therapy (RRT) is associated with the severity of AKI, blood vessel volume, electrolyte and acid–base state, urine volume, hemodynamics, and nutritional status. Therefore, listing RRT patients as AKI stage 3 might complicate AKI staging. Third, the confirmation of the baseline Scr value has important significance in the diagnosis of AKI using the 7-day window and the confirmation of stages. However, many patients do not have any record of a baseline Scr value; therefore, the incidence of AKI is always overestimated or underestimated.

Electrolyte disorder is a common complication of AKI. Changes in the blood magnesium level can cause tissue dysfunction in many organs and tissues, including the circulatory system, respiratory system, and muscles. The clinical incidence of hypomagnesemia in severe patients can reach 11–65% [15], whereas hypermagnesemia is relatively rare [16]. Therefore, there are many reports of the association between hypomagnesemia and mortality of severe patients, and there are fewer reports of the influence of serum magnesium ions on the prognosis of AKI patients [12, 17, 18]. Because related clinical symptoms caused by magnesium metabolism disorders are easily covered by other complications, it is necessary that clinical physicians monitor blood magnesium levels.

The incidence of malnutrition among elderly people is high, and malnutrition increases the incidence and mortality of AKI [19, 20]. Low serum albumin levels, low prealbumin levels, and low BMI are commonly used indicators for the evaluation of malnutrition in patients [21]. The statistical analysis results indicated that when BMI increases by 5.0 kg/m^2^, the in-hospital mortality rate and the all-cause mortality rate within 1 year also significantly increased accordingly [22]. However, many studies have shown that overweight and mild obesity in elderly people do not increase the all-cause mortality rate; in contrast, the overweight population has the lowest mortality rate [23–25]. One Japanese study on 263,940 elderly COPD patients aged > 65 years indicated that the all-cause in-hospital mortality rate of patients with overly low BMI was significantly increased. The result showed that compared to that of patients with a normal body weight, the mortality rate of patients with overweight and obesity was even lower. The mortalities of the underweight group, low-normal weight group (BMI 18.5–22.9 kg/m^2^), high-normal weight group (BMI 23.0–24.9 kg/m^2^), overweight group, and obesity groups were 14.3%, 7.3%, 4.9%, 4.3%, and 4.4%, respectively [23]. These study results suggest that the suitable BMI for elderly people might be higher than that for younger people.

Clinical studies have indicated that the prognosis of elderly AKI stage 1–2 patients mainly depends on a timely and effective diagnosis and treatment, and the prognosis of stage 3 AKI patients is usually poor. The progression of AKI stage indicates the increase in kidney damage level and aggravation of the disease. The abrupt increase in the Scr value within a short time might suggest the loss of more kidney function. Compared to a mild reduction of kidney function, more severe kidney function reduction suggests poor prognosis. Our data also showed that regardless of whether patients were in the 48-h diagnostic window or the 7-day diagnostic window group, the risk of death was higher when the AKI stage was higher. AKI stage 1 patients in this study accounted for nearly 50%. Although these patients had milder kidney function injury and the increased level of Scr was not high, their diagnosis could have been missed if Scr dynamic monitoring was not performed, thus progressing them to moderate and severe sustained AKI and increasing the development of poor prognosis. Therefore, the early prediction and early discovery of inducing factors are an indubitable responsibility of treating physicians. It has been shown that the mortality rate and dialysis requirement rate of AKI patients are positively correlated with the percentage of failure to consult nephrologists in time [26]. Nonnephrologists should fully understand the pathophysiological features of aging kidneys, control infection, be aware of drug nephrotoxicity, low blood volume, and principles of perioperative AKI prevention and treatment, raise the level of understanding and awareness of AKI, be familiar with the definition and diagnostic criteria of AKI, and invite nephrologist intervention in a timely manner, which is very important for improving the prognosis of elderly AKI patients.

Strengths of this study include the elderly age of the sample, the use of a consensus definition for AKI/CKD diagnosis, the 48-h and 7-day time windows of the KDIGO guidelines and stages, and baseline Scr being available in the entire sample of included patients. On the other hand, limitations of this study should be noted. (1) This was a single-center retrospective work, so the results may not be immediately applicable to other hospitalized patients. (2) The study excluded patients with CKD. As CKD is a potent risk factor for AKI and mortality, Therefore, biased results may be unavoidable. (3) The records of urine volume in many departments were not complete, and the AKI diagnostic criteria used only the Scr criteria; therefore, this might have caused missed diagnoses of AKI to some extent. (4) Factors that affected AKI prognosis were complicated, especially when the observation subjects were elderly people, with an average age of almost 87 years. They had many underlying diseases and poor general condition. Therefore, many comprehensive factors might affect the accuracy of prognosis analysis. (5) The study also did not explore the effects of the 48-h diagnostic window and the 7-day diagnostic window on renal outcome.

## Conclusions

The clinical differences of AKI and risk factors for 90-day mortality in elderly AKI individuals vary depending on the definition used. An increment of Scr ≥ 26.5 μmol/L in 48 h (48-h KDIGO window) alone predicts adverse clinical outcomes.
